# The efficacy of electrical stunning of New Zealand rock lobster (*Jasus edwardsii*) and freshwater crayfish (*Paranephrops zealandicus*) using the Crustastun™

**DOI:** 10.1017/awf.2023.76

**Published:** 2023-08-22

**Authors:** Nikki J Kells, Matthew Perrott, Craig B Johnson

**Affiliations:** 1 School of Veterinary Science, Massey University, Palmerston North, New Zealand; 2 Animal Welfare Science and Bioethics Centre, Massey University, Palmerston North, New Zealand

**Keywords:** animal welfare, Crustastun™, decapod crustacea, electrical stunning, neurophysiology, slaughter

## Abstract

Large numbers of decapod crustacea are farmed and harvested globally for human consumption. Growing evidence for the capacity of these animals to feel pain, and therefore to suffer, has led to increased concern for their welfare, including at slaughter. In New Zealand, decapod crustacea are protected by animal welfare legislation. There is a requirement that all farmed or commercially caught animals of these species killed for commercial purposes are first rendered insensible. The aim of this study was to evaluate the efficacy of the Crustastun™, a commercially available bench-top electrical stunner, in two commercially important New Zealand crustacean species; the rock lobster (*Jasus edwardsii*) and kōura (freshwater crayfish [*Paranephrops zealandicus*]). Animals were anaesthetised via intramuscular injection of lidocaine and instrumented to record the electrical activity of the nervous system, prior to being stunned according to the manufacturer’s instructions. Stunning efficacy was determined by analysing neural activity and observing behaviour post stunning. All ten *P. zealandicus* and three *J. edwardsii* appeared to be killed outright by the stun. Of the remaining *J. edwardsii*, six exhibited some degree of muscle tone and/or slow unco-ordinated movements of the limbs or mouthparts after stunning, although there was no recovery of spontaneous or evoked movements. One *J. edwardsii* was unable to be stunned successfully, likely due to its very large size (1.76 kg). None of the successfully stunned animals showed any evidence of return of awareness in the five minutes following stunning. It was concluded that the Crustastun™ is an acceptable method for killing *P. zealandicus* and for stunning all but the largest *J. edwardsii.*

## Introduction

The number of decapod crustaceans (crayfish, lobsters, prawns, and crabs) farmed for human consumption is growing, with recent estimates suggesting annual production to be in the hundreds of billions (http://fishcount.org.uk/fish-count-estimates-2/numbers-of-farmed-decapod-crustaceans). The inclusion of wild-caught animals is likely to significantly inflate these figures. Alongside consumption, concern for the welfare of these species has also grown in recent years as evidence of their capacity for sentience has emerged. There is now a large body of evidence indicating that decapod crustacea are capable of experiencing pain and anxiety-like states (Elwood 2019; Birch *et al*. 2021; Passantino *et al.* 2021).

This growing awareness and concern for crustacean welfare is reflected by legislation in countries such as Norway, Switzerland, New Zealand and, more recently, the United Kingdom, where the welfare of decapod crustacea is explicitly protected. In New Zealand, crustacean species including rock lobster (*Jasus edwardsii*), crabs and freshwater crayfish (*Paranephrops zealandicus*) are protected by the Animal Welfare Act (1999) and are acknowledged as being sentient (able to feel pain and distress). Regulations introduced in 2018 require that any farmed or caught animal of these species that are killed for commercial purposes must first be rendered insensible (Animal Welfare [Care and Procedures] Regulations 2018), for example by being stunned or chilled. This applies to primary processors as well as those slaughtering crustaceans at fish markets, and chefs and caterers slaughtering crustaceans in the kitchen (Animal Welfare [Care and Procedures] Regulations 2018).

The preferred method of making a crustacean insensible is through use of electrical current. Both chilling and electrical stunning are deemed acceptable means of rendering crustacean species insensible prior to killing/cooking. However, research suggests that cooling may only induce paralysis, as opposed to insensibility (Yue 2008; Weineck *et al.* 2018), meaning that perceptual awareness may be retained until the animals are killed by a secondary method such as cooking. Electrophysiological recordings demonstrated that crayfish and lobsters cooled to 0˚C in an ice slurry exhibited only minor reductions in spontaneous neural activity, and evoked neural responses to mechanical and electrical stimuli were still present after 60-min chill time (Fregin & Bickmeyer 2016). Furthermore, the time to loss of neural activity after transfer into boiling water was delayed relative to non-chilled controls. Chilling to 0˚C has also been shown to be ineffective for stunning the Australian giant crab (*Pseudocarcinus gigas*) (Gardner 1997) or the blue crab (*Portunus pelagicus*) (Roth & Øines 2010). In contrast, electrical stunning using an appropriate charge has been shown to effectively stun or kill several species of crab, crayfish, and lobster within seconds of application (Ogawa *et al.* 2007; Albalat *et al.* 2022; Fregin & Bickmeyer 2016; Weineck *et al.* 2018; Neil *et al*. 2022), making this a more humane choice.

At least one electrical stunning device that is suitable in a commercial kitchen setting is commercially available (Crustastun™, Mitchell and Cooper Ltd, UK). The Crustastun™ is a compact bench-top electrical stunning device designed to administer a lethal electric shock to shellfish such as lobsters and crabs prior to cooking by administering a 110 V, 2–5 amp charge for a duration of 5 s (lobsters) or 10 s (crabs). It was specifically developed in the UK to stun North Atlantic/European species of lobster and crabs. Published results indicate that the device is effective in stunning European lobsters (*Homarus gammarus*), langoustines (*Nephrops norvegicus*), and brown crabs (*Cancer pagurus*) (Albalat *et al.* 2022; Neil 2022), ensuring no recovery before death.

New Zealand has two common species of rock lobster: the red or Southern rock lobster (*J. edwardsii*) and the green or packhorse rock lobster (*Sagmariasus verreauxi*). The red rock lobster is the most valuable inshore commercial fishery in New Zealand, producing 2,500–3,000 tonnes annually, worth NZ$340 million in 2022 (Seafood New Zealand 2022). Freshwater crayfish (*P. zealandicus*), known locally as kōura, are common in many parts of New Zealand and are a culturally significant species. Whilst currently of low commercial significance, with only ~100 tonnes produced annually for the domestic market (Hollows 2020), there is the potential for kōura aquaculture to become an important export industry in the future. The suitability of this device for stunning commercially important New Zealand crustacean species has not been previously evaluated. *J. edwardsii* can reach up to 60 cm in length and 8 kg in weight at maturity, although commercially harvested animals are more likely to be in the range of 0.6–1.0 kg (D Sykes, New Zealand Rock Lobster Industry Council, personal communication 2020). Potential size differences between NZ rock lobster and North Atlantic/European lobster species at harvesting raise concerns that available devices may not stun them effectively due to differences in tissue depth/thickness, structure and neurological architecture, e.g. number and location of the heart and ganglia may vary between European and New Zealand species. Currently there are no data on the effectiveness of commercially available stunning devices on any New Zealand crustacean.

Electrophysiological tools can be used to monitor central and peripheral nervous system activity. In birds, mammals and fish, measures of spontaneous or evoked neural activity in the brain have been used to assess sensibility and/or death in response to different stunning and slaughter methods (Martin *et al.* 2016; Verhoeven *et al*. 2016; Bowman *et al*. 2019; Rault *et al.* 2020). The absence of spontaneous or evoked brain activity, or the presence of epileptiform seizures following application of an electric current, are both considered incompatible with conscious awareness.

Due to the absence of a centralised brain, EEG recording cannot be used to assess sensibility in lobsters or crayfish. In the few reported studies, researchers have utilised various electrophysiological techniques to measure spontaneous or evoked neural activity in different regions of the nervous system as indicators of insensibility and/or death after stunning. These include recording neural signal propagation between adjacent abdominal ganglia in response to mechanosensory stimulation (Fregin & Bickmeyer 2016), recording spontaneous and evoked neural activity in the cardiac ganglion and skeletal muscles (Weineck *et al*. 2018), and measuring intrinsic and evoked activity in exposed nerve preparations (Neil 2012). In all cases, recordings were made using electrodes that were directly implanted into target nerves which either required the removal of the epidermis and dissection of the covering muscles (Neil 2012; Fregin & Bickmeyer 2016) or for holes to be drilled through the carapace above target nerves (Weineck *et al.* 2018). In contrast with procedures used for surgical implantation of electrodes in mammals, instrumentation was performed without anaesthesia, potentially resulting in the experience of pain or other negative sensations.

The objective of this study was to evaluate the efficacy of the Crustastun™ in two commercially important New Zealand crustacean species: the rock lobster (*J. edwardsii*) and freshwater crayfish (*P. zealandicus*). Data obtained from this study will contribute to developing regulations, standards and guidelines and mitigating risks in compliance. Additionally, confirmation of the efficacy of these devices in New Zealand species will create certainty for operators who may wish to purchase and use them.

## Material and methods

### Ethical approval

This study was undertaken with the approval of the Massey University Animal Ethics Committee (protocol # 20/41) and carried out in accordance with the Massey University Code of Ethical Conduct for the Use of Animals in Research, Teaching and Testing.

### Study animals

Twenty animals in total were used in the research, including ten New Zealand rock lobster (*J. edwardsii*) and ten kōura, or freshwater crayfish (*P. zealandicus*).

*J. edwardsii* were sourced from the southern fishery (Whitecaps Fishing Company, Stewart Island, New Zealand) and were requested to be at the upper end of the size of commercially harvested animals (generally 0.6–1.2 kg). Their mean weight was 981 g (range: 721–1,760 g), with a mean carapace length of 120.6 mm (range: 109.5–153 mm). They were caught off the coast of Fiordland and transported overnight in two separate batches in polystyrene boxes to Massey University from Stewart Island. The polystyrene transport boxes were lined with ice packs and the animals kept separate from the ice packs and each other with wet paper and sacking. The first six animals arrived on 6th October and the remaining four on 18th November 2020.

*P. zealandicus* were sourced commercially (Ernslaw One Ltd, Tapanui, New Zealand) and were transported overnight in a polystyrene box lined with ice packs, separated by wet sacking and wood-shavings. They had a mean weight of 44.8 g (range:28.0–61.0 g), and a mean carapace length of 53.4 mm (range: 45.5–63.1 mm). All ten animals arrived on 10th November 2020.

All animals were judged to be healthy on arrival, based on clinical examination by an experienced veterinarian, and were acclimatised for a minimum of one week prior to use in the study. Both species were held in circulating cold water systems. *J. edwardsii* were held in individual 40-L holding tanks filled with natural seawater and topped up with artificial seawater. Aerated seawater was circulated at a constant rate of 1–2 L per minute. The main reticulated supply contained an in-tank biofilter. The holding room was maintained at 14°C and external windows provided a natural light/dark cycle. Animals were fed greenshell lipped mussels (*Perna canaliculus*) daily or every second day, based on consumption rate. Tanks were vacuumed twice weekly to remove faeces and detritus. *P. zealandicus* were housed together in a single 50-L aquarium located in a room-temperature laboratory. The tank was filled with stream water and topped up with purified water, and it contained stones and logs to provide shelter and separation of individuals. Tank water was chilled to approximately 13°C and aerated. Animals were fed dried mealworms and/or blood worms daily or every second day. The tank was vacuumed weekly along with a partial water change to remove nitrogenous wastes. All animals were at the intermoult stage at the time of harvest and testing.

At the conclusion of data collection, individual bodyweight (g) was measured using a digital scale (2 dp precision), and carapace length and tail width (mm) were measured using digital calipers (2 dp precision).

### Experimental protocol

#### Pilot study

Pilot experimental studies were undertaken utilising two *J. edwardsii* for the purpose of determining the optimal anaesthesia protocol and electrode placement. The animals were transported individually to the Neuroscience Laboratory in thermally insulated containers with shallow seawater. It was decided to anaesthetise the animals prior to electrode placement, to prevent the experience of pain or other negative sensations. As there was little information available on effective analgesia for decapod crustacea, intramuscular injection of lidocaine was selected after consultation with a veterinary anaesthetist. Anaesthesia was induced via intramuscular injection of 2% lidocaine hydrochloride (Lopaine, Ethical Agents, New Zealand), injected into the musculature of the first abdominal segment. The anaesthetic dose used for the two pilot animals, along with their weight and size, is provided in Table 1.

**Table 1.** Size, weight, and anaesthetic doses for *J. edwardsii* (n = 10) and *P. zealandicus* (n = 10) used to evaluate the efficacy of the Crustastun™ bench-top electrical stunner

Animal ID	Carapace length (mm)	Tail width^1^ (mm)	Lidocaine dose (mg)	Weight (g)	Lidocaine (mg kg^–1^)	Outcome of stun
*J. edwardsii* 1^a^	113	64	100	776	128.9	Killed
*J.edwardsii* 2^a^	111	60	60	723	83	Killed
*J.edwardsii* 3	117	72	116	1163	99.7	Killed
*J.edwardsii* 4	116	65	90	899	100.11	Stunned
*J.edwardsii* 5	115	65	81	814	99.5	Stunned
*J.edwardsii* 6	153	81	176	1760	100	Failed^2^
*J.edwardsii* 7	120	65	94	943	99.7	Stunned
*J.edwardsii* 8	121	66	88	877	100.3	Stunned
*J.edwardsii* 9	110	65	72	721	99.7	Stunned
*J.edwardsii* 10	131	76	112	1130	99.1	Stunned
*P. zealandicus* 1	51	29	5^b^	44	113.6	Killed
*P. zealandicus* 2	52	25	15^c^	43	348.8	Killed
*P. zealandicus* 3	47	24	8	28	285.7	Killed
*P. zealandicus* 4	56	29	10	48	208.3	Killed
*P. zealandicus* 5	58	32	10	51	196.1	Killed
*P. zealandicus* 6	53	29	9	44	204.6	Killed
*P. zealandicus* 7	63	34	12	61	196.7	Killed
*P. zealandicus* 8	46	23	6	28	214.3	Killed
*P. zealandicus* 9	54	31	11	54	203.7	Killed
*P. zealandicus* 10	55	31	10	47	212.8	Killed

1 Distance between primary spines on second abdominal segment

2 Animal was too large to properly place in stunner and was unable to be stunned

a Indicates animals that were part of the pilot study to determine appropriate anaesthetic dose and electrode configuration

b Individual showed signs of anaesthetic wearing off due to delay in fixing electrodes

c Lidocaine given in 3 × 5 mg increments to effect prior to instrumentation

The first animal (*J. edwardsii* 1) was given 5 mL of 2% lidocaine (100 mg) as a single dose. Within 5 min of administration, the animal was deemed effectively anaesthetised, based on relaxation of the tail muscle, absence of muscle tone in the legs, and loss of righting reflex. In the second animal (*J. edwardsii* 2), 60 mg lidocaine was given incrementally in 1 mL doses (20 mg). After each 20 mg dose, the animal was returned to the holding box for 10 min, after which time sedation was assessed. After the third dose (60 mg total), the animal showed loss of muscle tone in the tail and legs and minimal righting reflex, so was instrumented and stunned.

**Figure 1.**
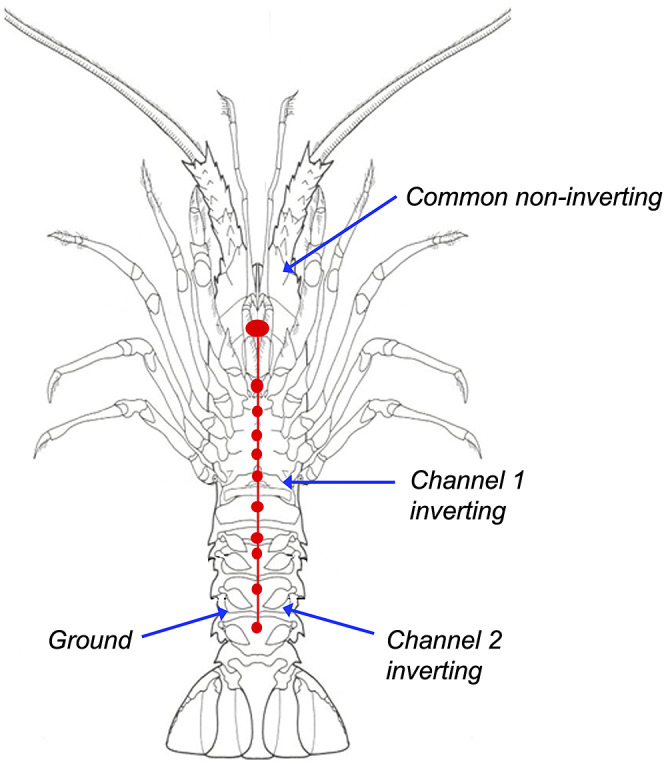
Ventral view of a rock lobster showing the approximate location of the central nerve cord and associated ganglia (red) and illustrating the sites of electrode placement (blue arrows) used for recording electrical activity from the nervous system of animals undergoing electrical stunning.

Following induction of anaesthesia, animals were instrumented for electrical recording. A four-electrode montage was used to record two channels of electrical activity: the first between the head and cranial abdomen, incorporating the cerebral ganglion (also referred to as the suboesophageal ganglion) and all five thoracic ganglia; the second from the head to caudal abdomen, incorporating the cerebral ganglion, thoracic ganglia, and first four abdominal ganglia (Figure 1). Subcutaneous 27-gauge, 0.5-inch stainless steel needle electrodes (Ambu, Ballerup, Denmark) were inserted between carapace segments into the soft tissues as indicated in Table 2 (note different placement of the inverting electrodes between animals 1 and 2). Electrodes and leads were secured to the animal and isolated from the environment by application of cyanoacrylate adhesive (Super ‘T’ gap filling, Satellite City, CA, USA) and accelerant (Zip Kicker, Zapglue, Illinois, USA). Electrical activity was recorded using IsoDAM signal amplifiers (WPI Instruments, FL, USA), digitised using an analogue to digital converter (Powerlab, ADI, New Zealand) and recorded to computer file (Chart software, ADI, New Zealand).

**Table 2.** Placement of recording electrodes used to record electrical activity from *J. edwardsii* (n = 10) and *P. zealandicus* (n = 10) subject to electrical stunning using the Crustastun™ bench-top electrical stunner

Animals	Common non-inverting	Channel 1 inverting	Channel 2 inverting	Ground
*J.edwardsii* 1 (pilot)	Articulation of left antenna with head	Lateral under carapace of first abdominal segment on left	Lateral under carapace of fourth abdominal segment on left	Lateral under carapace of fourth abdominal segment on right
*J.edwardsii* 2 (pilot)	Articulation of left antenna with head	Dorsal midline between first and second abdominal segment	Dorsal midline between fourth and fifth abdominal segment	Lateral under carapace of fourth abdominal segment on right
*J. edwardsii* 3–10 *P. zealandicus* 1–10 (main study)	Articulation of left antenna with head	Lateral under carapace of first abdominal segment on left	Lateral under carapace of fourth abdominal segment on left	Lateral under carapace of fourth abdominal segment on right

Following electrode placement, three minutes of electrical activity was recorded as a baseline after which animals were placed in the stunning device (Crustastun™, Mitchell and Cooper, UK). The chamber of the Crustastun™ measured 440 × 360 × 130 mm (length × width × depth). The stun was delivered using setting 2 (Lobster; 110 V, 50 Hz for 5 s). Delivery of a successful stun current was illustrated by the stun current indicator on the front panel of the device. Where the stun current was too low or high, this was also illustrated by the stun current indicator and accompanied by an audible alarm. Electrical data were continuously recorded during the delivery of the stun and for a 5-min period afterwards. The animal was then removed from the stunning device and placed in a separate container. Stunning was deemed successful if the animal was quiescent upon removal from the stunning device. Animals were observed for 20–30 min after removal from the stunner to determine whether they had been killed outright by the stun. Death was ascertained by permanent loss of muscle tone and absence of spontaneous or evoked movement of the body or appendages (including mouthparts and pleopods).

The electrode placement used in *J. edwardsii* 1 (Table 2, Figure 1) was subsequently adopted for the main study because that used in *J. edwardsii* 2 was associated with obvious burning of the carapace during stunning. This was likely due to contact between the recording electrodes and the stunner plate that did not occur with the configuration used for *J. edwardsii* 1. Both electrode positions allowed adequate electrical activity to be recorded. Examination of electrophysiological recordings from the two pilot animals revealed no observable differences in the amplitude of baseline (pre-stunning) data, suggesting that the higher dose of lidocaine given to animal 1 did not interfere with data acquisition. In mammals, increasing depth of anaesthesia is known to influence brain electrical activity, therefore the minimum dose required for effective anaesthesia is adopted for studies of brain activity. Based on the apparent lack of impact on electrophysiological recordings, an anaesthetic dose of 100 mg kg^–1^ lidocaine was adopted for the main study (Table 1).

The results of the pilot study were subsequently found to be comparable with those from the main study and so these two animals were included in the final dataset.

#### Main study

The remaining four *J. edwardsii* from the initial batch (received on October 6th) were tested on October 16th 2020. The four animals from batch 2 (received on November 18th), were tested on December 2nd 2020. On the day of testing, animals were individually transported to the Neuroscience Laboratory (a 5-min walk) in thermally insulated containers with shallow seawater. Each animal was anaesthetised by intramuscular injection of 2% lidocaine hydrochloride at a dose rate of 100 mg kg^–1^, injected into the musculature of the first abdominal segment. Following injection, the animal was placed back in the transport cage and left undisturbed for 10 min to allow anaesthesia to take effect. See Table 1 for weight and size of crayfish and dose of anaesthetic administered.

Once anaesthetised, recording electrodes were applied (as described in Table 2) and data were recorded as described for the pilot study above. Any animals for which death by stunning was not confirmed were subsequently euthanased via intramuscular injection of 2.5 g of pentobarbital potassium (Provet NZ Pty Ltd, New Zealand), followed by midline separation using a sharp knife.

All ten *P. zealandicus* were tested on November 10th 2020. Animals were removed individually from the home tank (located in the Neurophysiology Laboratory), weighed and lightly anaesthetised via intramuscular injection of lidocaine. The animal was then covered with a freshwater-dampened towel and left undisturbed for 10 min to allow anaesthesia to take effect. A dose rate of approximately 200 mg kg^–1^ (see Table 1) was required to achieve adequate anaesthesia (cessation of purposeful movement and reduced or absent muscle tone in the tail) for the duration of electrode placement prior to stunning. Animals were then instrumented for electrical data recording as described for *J. edwardsii* above (refer to Table 2 for electrode placement). Due to their smaller size, and in anticipation of likely commercial conditions, *P. Zealandicus* were stunned in pairs.

#### Data analysis

Raw data were subjected to Fast Fourier transformation, yielding the summary variables median frequency (F50), 95% spectral edge frequency (F95) and total power (PTOT) for consecutive 1-s epochs. The mean F50, F95 and PTOT were calculated for the 60 s immediately prior to stunning and 60 s immediately after stun application for each channel in each individual. From this, the mean changes in F50, F95 and PTOT were determined for the entire cohort. Any data-points contaminated with movement artefact (e.g. spontaneous tail or limb movements, closure of stunner lid) were excluded from analysis. This meant that some data sets included less than 60 EEG epochs per period.

Paired *t*-tests were performed to compare post-stunning means for each frequency variable with pre-stunning values. Frequency distributions of the paired differences for each variable were calculated to ensure that the assumptions for using the paired sample *t*-test were met (normal distributions with no outliers). Statistical analyses were conducted in SAS v9.4 (SAS Institute Inc, Cary, NC, USA).

## Results

### Responses to stunning

Regarding *J. edwardsii* (with the exception of one animal; #6, weighing 1,760 g), all individuals were delivered a successful stun (as indicated by the stunning device). In the case of animal #6, three attempts to deliver a successful stun resulted in short bursts of current (~2 s per attempt) followed by a stun failure alarm. When the lid was opened, the animal was exhibiting purposeful movements comparable to those observed prior to being placed in the stunner. Of the other nine, three appeared to be killed outright by the stun (no evidence of movement or muscle tone). Six exhibited some degree of muscle tone and/or occasional very slow unco-ordinated movement of the mouthparts, pleopods, or limbs. Such movements or muscle tone did not resemble those observed prior to anaesthesia, or subsequent to anaesthesia and prior to stunning. No recovery of spontaneous or evoked movement (reflex response to eye stalk touch) was observed in any animal over a period of up to 60 min subsequent to stunning. As a precaution, the animals for which outright death was not certain were euthanased with pentobarbital followed by midline separation (for details, see *Materials and methods*).

All ten *P. zealandicus* appeared to be killed outright by the stun (no evidence of spontaneous or evoked movement, or muscle tone after removal from the stunner).

### Electrophysiological data

Data from nine *J. edwardsii* were included in the analyses. Of note, there appeared to be more individual variability in the electrical responses of *J. edwardsii* to stunning, when compared with *P. zealandicus.* This may have been due to the larger variability in size and weight of individuals (Table 1). The differences between pairs (pre and post stun) for both species were found to follow an approximately normal distribution (skewness and kurtosis scores within the range –2 to +2) for all three dependent variables (F50, F95, PTOT).

In *J. edwardsii,* data from Channel 1 demonstrated a consistent pattern of decreases in F50 and F95 and an increase in PTOT following stunning (Table 3). Of the three variables, only F50 was significantly different post stunning (t[8] = 2.32; *P* =0.049). Data from Channel 2 were less consistent, with some individuals showing the same pattern of decreases in F50 and an increase in PTOT as seen in Channel 1, whereas little to no change was observed in others. Whilst a similar trend was observed overall, high individual variability meant these changes were less marked and did not reach statistical significance (Table 3). There were no apparent differences in data between individuals that were or were not deemed to have been killed outright by the stun.

**Table 3.** Results of statistical analyses of electrophysiological data recorded from J. edwardsii (n = 9) in the 60 s prior (pre) and 60 s immediately after (post) electrical stunning using the Crustastun™ commercial bench-top stunner. Data are presented as least square mean (SEM)

Variable	Pre	Post	*t*-value	DF	*P*-value
Channel 1^1^					
Median frequency (F50)	11.56	9.36	2.32	8	0.048
	(0.68)	(0.86)			
Spectral edge frequency (F95)	27.78	27.25	2.06	8	0.073
	(0.13)	(0.20)			
Total power (P_TOT_)	2.86	4.04	−1.94	8	0.088
	(0.40)	(0.37)			
Channel 2^2^					
Median frequency (F50)	7.36	6.28	1.25	4	0.278
	(0.56)	(0.37)			
Spectral edge frequency (F95)	26.91	26.52	0.89	4	0.422
	(0.19)	(0.22)			
Total power (P_TOT_)	2.13	2.70	-2.06	4	0.105
	(0.20)	(0.26)			

1 Recorded between head and cranial abdomen

2 Recorded between head and caudal abdomen

A typical example of changes in the frequency spectra after stunning is shown in Figure 3 where a marked increase in power in the lower frequencies is clearly visible.

**Figure 2.**
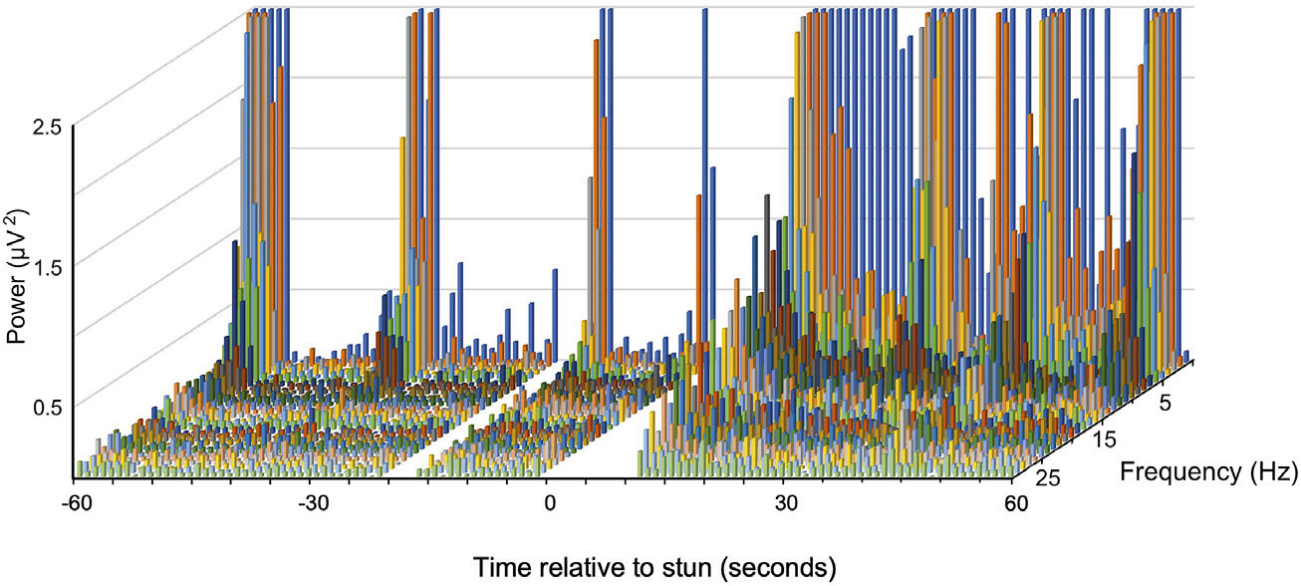
Example of a compressed spectral array from an individual *J. edwardsii* (#10) before and after electrical stunning (Time 0) using the Crustastun™ commercial bench-top stunner. Recorded from electrodes spanning the head to cranial abdomen (Channel 1). Missing data are where artefact induced by transfer to the stunner and/or stun application have been removed.

**Figure 3.**
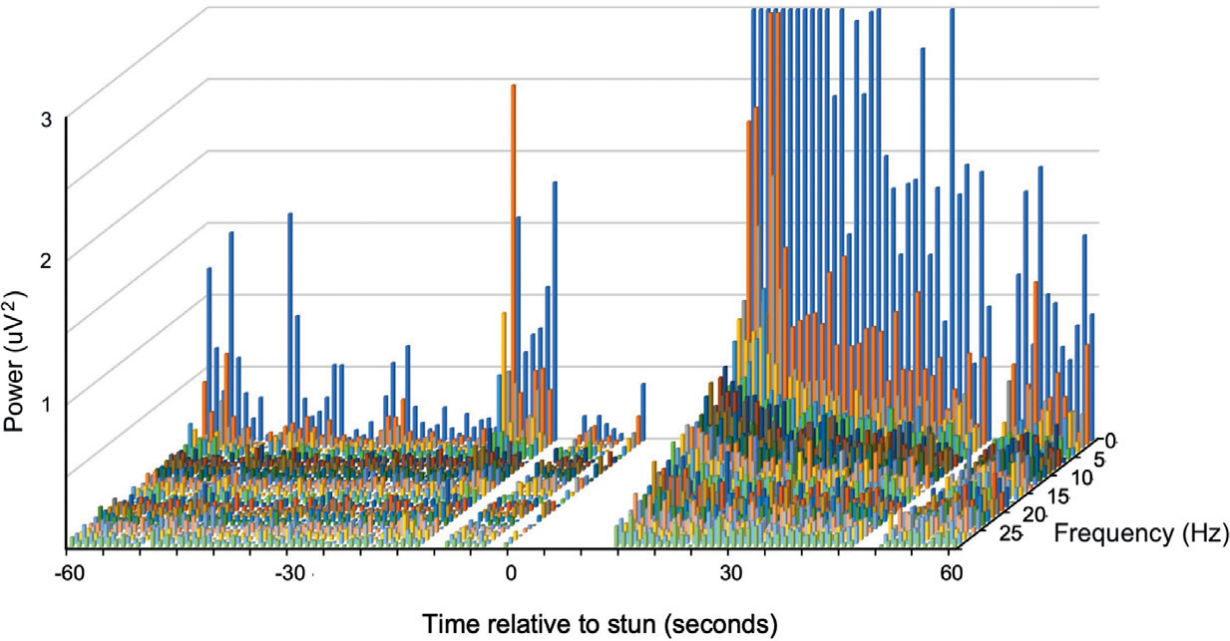
Example of a compressed spectral array from an individual *P. zealandicus* (#7) before and after electrical stunning (Time 0) using the Crustastun™ commercial bench-top stunner. Recorded from electrodes spanning the head to cranial abdomen (Channel 1). Missing data are where artefact induced by transfer to the stunner and/or stun application have been removed.

In *P. zealandicus*, data from Channel 1 demonstrated a consistent pattern of decreases in F50 and F95 and an increase in PTOT following stunning (Table 4). All three variables differed significantly from baseline post stunning. Data from Channel 2 were less consistent, with some individuals showing the same pattern of decreases in F50 and an increase in PTOT as seen in Channel 1, whereas little to no change was observed in others. Whilst a similar trend was observed overall, high individual variability meant these changes were less marked (Table 4).

**Table 4.** Results of statistical analyses of electrophysiological data recorded from P. zealandicus (n = 10) in the 60 s prior (pre) and 60 s immediately after (post) electrical stunning using the Crustastun™ commercial bench-top stunner. Data are presented as least square mean (SEM)

Variable	Pre	Post	t-value	DF	*P*-value
Channel 1^1^					
Median frequency (F50)	12.15	7.96	9.06	9	<0.001
	(0.38)	(0.32)			
Spectral edge frequency (F95)	27.86	27.74	4.06	9	0.003
	(0.11)	(0.11)			
Total power (P_TOT_)	2.26	4.46	–9.03	9	<0.001
	(0.19)	(0.15)			
Channel 2^2^					
Median frequency (F50)	13.81	12.07	2.72	9	0.024
	(0.28)	(0.84)			
Spectral edge frequency (F95)	28.09	27.75	2.58	9	0.03
	(0.11)	(0.23)			
Total power (P_TOT_)	1.53	2.10	–1.17	9	0.272
	(0.24)	(0.73)			

1 Recorded between head and cranial abdomen

2 Recorded between head and caudal abdomen

Despite this, both F50 and F95 differed significantly from baseline post stunning.

A typical example of changes in the frequency spectra after stunning is shown in Figure 2, where the marked increase in power in the lower frequencies is clearly visible.

## Discussion

This study aimed to evaluate the ability of the Crustastun™ to adequately stun and/or slaughter *J. edwardsii* and *P. zealandicus* for commercial purposes. The criteria for a successful stun were that insensibility would be achieved rapidly (within 1–2 s) and maintained for long enough to allow killing by conventional means if the animals were not killed by the stun itself. To our knowledge, this is the first study that has attempted to measure global neural electrical activity as an indicator of awareness following electrical stunning. Previous studies have utilised electrodes implanted in target nerve fibres to assess nerve signal propagation in response to mechanical (Fregin & Bickmeyer 2016; Weineck *et al.* 2018) or electrical (Fregin & Bickmeyer 2016) stimuli as indicators of cessation of awareness after stunning/slaughter. Whilst providing valuable information on neural activity and potential awareness, such methods are not without limitations. The process of electrode implantation is invasive, requiring carapace removal or local destruction, along with displacement of organs and/or dissection of the covering muscles to expose the target nerves. Such procedures, when performed without anaesthesia, are likely to cause pain. Furthermore, the absence of sedation or general anaesthesia requires the animal to be restrained during recording to reduce movement artefact and prevent electrode displacement. In the case of the work by Neil (2012, 2022), whilst both intrinsic and evoked activity were evaluated after stunning, these consisted of extracellular recordings carried out on the isolated head, thorax, or limbs rather than intact animals, raising concerns about animal welfare and the generalisability of results to intact animals.

In order to record nervous system activity from intact animals in a minimally invasive manner that did not require prolonged recovery between instrumentation and testing, we developed a method to record spontaneous electrical activity using electrodes inserted into the musculature through naturally occurring gaps between adjacent carapace segments. To safeguard their welfare, animals were anaesthetised with lidocaine prior to electrode placement and subsequent stunning. Lidocaine has previously been used to induce general anaesthesia in freshwater crayfish (*Orconectes virillis*) (Brown *et al.* 1996). A dose of 400 mg kg^–1^ lidocaine injected into the tail muscle induced a state of general anaesthesia within 1.5 min, for a duration of 25 min (Brown *et al.* 1996). In our study, lidocaine doses ranging from 200–350 mg kg^–1^ appeared to induce general anaesthesia in *P. zealandicus* for a duration sufficient to enable instrumentation and stunning. There are no previous reports of lidocaine being used for general anaesthesia in lobster species. However, a dose of approximately 100 mg kg^–1^ appeared sufficient to induce a state of light general anaesthesia in *J. edwardsii* in this study.

Of the nine *J. edwardsii* to which a stun was successfully applied, stunning with the Crustastun™ resulted in instantaneous changes to the electrical activity of the nervous system that qualitatively resembled EEG changes seen in mammals during application of stunning techniques known to cause insensibility (Johnson *et al.* 2012; Rault *et al.* 2014; Sabow *et al.* 2017). This is consistent with data from European crayfish (*A. astacu*s, *A. leptodactilus*) and American lobsters *(H. gammarus),* where epileptiform seizures were reported in response to stunning with the Crustastun™ (Fregin & Bickmeyer 2016).

Three *J. edwardsii* appeared to have been killed outright by the stun with no sign of return of behavioural function following the stun. Those that were not apparently killed outright showed no sign of return of normal behaviour following the stun (purposeful movements or response to mechanical stimulation), but it was not possible to determine whether this was due to the stun or to continuation of the effects of the anaesthetic. Based on reports of the short-term nature of lidocaine anaesthesia in crayfish (Brown *et al.* 1996), it may be that the prolonged behavioural quiescence post stunning was due to the effects of stunning itself. In the present study, the effects of IM lidocaine also appeared to be relatively short-lived. This was especially notable in *P. zealandicus*, where a 5 mg dose (~100 mg kg^-1^) appeared to give ~5 min of sedation. Regardless, the failure to include an anaesthesia-only control group in the present study limits data interpretation and should be addressed in future studies.

Recordings from the head to cranial abdomen region appear more sensitive than those from the head to caudal abdomen for detecting changes in neural activity after stunning. Whilst the reason for this finding is not clear, it suggests that the former configuration alone is sufficient for observing changes in neural activity in these species.

It should be noted that there were no apparent differences between the electrophysiological data from individuals that were or were not deemed to be killed outright. It may be that the duration of recording (5 min post stunning) did not allow time for such changes to emerge, or that the movements observed post stunning in some individuals were an artefact of the stun itself and these animals were in fact killed by the stun. Further exploration of this methodology in non-anaesthetised animals would help to unpack these responses.

Previous studies have reported that electrical stunning with the Crustastun™ induced outright death in European lobsters (Neil 2012) and langoustines (Albalat *et al.* 2022). In contrast, Fregin and Bickmeyer (2016), reported recovery of function approximately 18 h after stunning of European crayfish and American lobster. Similarly, electrical stunning of red swamp crayfish (60V 120 Hz, 10 s) induced paralysis but not death (Weineck *et al.* 2018). The reason for the discrepancies between these studies, and between individuals in the present study, is unclear.

The largest animal in the *J. edwardsii* group was 30% longer and 96% heavier than the mean of the other nine animals, weighing 1,760 g (Table 1). It was physically difficult to fit into the stunner, needing to have its tail folded underneath it rather than being placed flat on the plate (the total length of this animal with antennae folded back was 470 mm; the inside of the stunning tank measured 440 × 360 mm; length × width). During stunning, the Crustastun™ detected that the animal was unsuitable and did not deliver a stun. This may have been due to the position of the animal resulting in an electrical load that was outside the range of the device or may have been due to poor contact between the animal and the electrodes, or to displacement of the water in the stunner raising the level and shorting the electrodes. This animal was exceptionally large by restaurant standards – the New Zealand restaurant trade typically deals with lobster live weights in the range of 600–1,000 g (D Sykes, New Zealand Rock Lobster Industry Council, personal communication 2020).

In all ten *P. zealandicus* used in this study, stunning with the Crustastun™ resulted in instantaneous changes to the electrical activity of the nervous system that qualitatively resembled EEG changes seen in mammals during the application of stunning techniques (Johnson *et al.* 2012; Rault *et al.* 2014; Sabow *et al.* 2017). All ten animals appeared to have been killed outright by the stun with no sign of return of behavioural function following the stun.

All animals used in this study were anaesthetised throughout and were either killed by the stun or killed prior to the return of normal behaviour. The anaesthetic was considered necessary to guard the welfare of the animals because this device has not previously been used on the two species in question. However, this meant that the duration of the stun in those *J. edwardsii* that were apparently not killed outright could not be determined. Electrical activity was recorded for 5 min following the stun and there was no evidence of return of activity that might indicate awareness during this time. These results suggest that the maximum stun-to-kill time for animals that are not killed outright is at least 5 min, though it could be much longer. Studies in European crayfish and American lobster suggest that the effective stun duration could be 10 min or greater in these species (Neil 2012; Fregin & Bickmeyer 2016). Further research is necessary to accurately determine a safe maximum stun-to-kill time for non-anaesthetised *J. edwardsii.*

There is little information available regarding appropriate methods for humanely killing decapod crustaceans. Midline separation, causing immediate disruption of all ganglia, is deemed an effective means of euthanasia, but is permissible only after induction of sedation or general anaesthesia (Leary *et al.* 2020). In this study, we elected to administer a high dose of potassium pentobarbital prior to midline separation in rock lobster for which death by stunning could not be confirmed. Pentobarbital was chosen based on its efficacy as an anaesthetic and euthanasia agent in a variety of species, including fish, despite there being no previous reports of its use in lobster. In the one rock lobster where electrical stunning failed, potassium pentobarbital injection induced rapid behavioural quiescence consistent with sedation or anaesthesia prior to separation. No attempt was made to determine anaesthesia depth or duration, nor whether pentobarbital alone was sufficient to cause death.

### Animal welfare implications

The electrophysiological data collected using the non-invasive methodology adopted in this study appears to be useful for identifying changes in spontaneous neural electrical activity in response to electrical stunning in NZ rock lobster (*J. edwardsii*) and freshwater crayfish (*P. zealandicus*). The observed increase in total power observed after stunning was broadly consistent with that seen in mammals, where the presence of epileptiform seizures (deemed incompatible with conscious awareness) results in corresponding increases in total power. The absence of observable differences in the frequency spectrum of those animals for which outright death could not be confirmed suggests that behavioural indicators may be more reliable for ascertaining death.

The Crustastun™ device appears to be an acceptable method of killing or adequately stunning all but the largest *J. edwardsii.* No stun was delivered to the largest animal, presumably because the electrical load of this animal fell outside of the device’s programmed range. This study is not able to specify a cut-off point beyond a liveweight of 1,160 g for effective stunning of *J. Edwardsii.* The results do however show the need for the tail to lie flat and for the animal to be fully enclosed in order for the stunner to operate reliably, meaning animals that exceed 440 mm in length (with antennae folded back) are not suitable for stunning using the device. The Crustastun™ appears to be an acceptable method of killing *P. zealandicus.* Further research is required to validate the stunner in species of interest under commercial conditions.
